# A Novel Hierarchical Deep Learning Framework for Diagnosing Multiple Visual Impairment Diseases in the Clinical Environment

**DOI:** 10.3389/fmed.2021.654696

**Published:** 2021-06-07

**Authors:** Jiaxu Hong, Xiaoqing Liu, Youwen Guo, Hao Gu, Lei Gu, Jianjiang Xu, Yi Lu, Xinghuai Sun, Zhengqiang Ye, Jian Liu, Brock A. Peters, Jason Chen

**Affiliations:** ^1^Department of Ophthalmology and Visual Science, Eye, and Ear, Nose, and Thorat Hospital, Shanghai Medical, College Fudan University, Shanghai, China; ^2^Department of Ophthalmology, Affiliated Hospital of Guizhou Medical University, Guiyang, China; ^3^Key Laboratory of Myopia, Ministry of Health (Fudan University), Shanghai, China; ^4^Shanghai Engineering Research Center of Synthetic Immunology, Fudan University, Shanghai, China; ^5^AI Laboratory, Deepwise Healthcare, Beijing, China; ^6^Wuhan Servicebio Technology, Wuhan, China; ^7^Epigenetics Laboratory, Max Planck Institute for Heart and Lung Research, Bad Nauheim, Germany; ^8^Cardiopulmonary Institute (CPI), Bad Nauheim, Germany; ^9^Complete Genomics Inc., San Jose, CA, United States

**Keywords:** artificial intelligence, hierarchical deep learning framework, visual impairment disease, coarse-to-fine, multi-task multi-label

## Abstract

Early detection and treatment of visual impairment diseases are critical and integral to combating avoidable blindness. To enable this, artificial intelligence–based disease identification approaches are vital for visual impairment diseases, especially for people living in areas with a few ophthalmologists. In this study, we demonstrated the identification of a large variety of visual impairment diseases using a coarse-to-fine approach. We designed a hierarchical deep learning network, which is composed of a family of multi-task & multi-label learning classifiers representing different levels of eye diseases derived from a predefined hierarchical eye disease taxonomy. A multi-level disease–guided loss function was proposed to learn the fine-grained variability of eye disease features. The proposed framework was trained for both ocular surface and retinal images, independently. The training dataset comprised 7,100 clinical images from 1,600 patients with 100 diseases. To show the feasibility of the proposed framework, we demonstrated eye disease identification on the first two levels of the eye disease taxonomy, namely 7 ocular diseases with 4 ocular surface diseases and 3 retinal fundus diseases in level 1 and 17 subclasses with 9 ocular surface diseases and 8 retinal fundus diseases in level 2. The proposed framework is flexible and extensible, which can be inherently trained on more levels with sufficient training data for each subtype diseases (e.g., the 17 classes of level 2 include 100 subtype diseases defined as level 3 diseases). The performance of the proposed framework was evaluated against 40 board-certified ophthalmologists on clinical cases with various visual impairment diseases and showed that the proposed framework had high sensitivity and specificity with the area under the receiver operating characteristic curve ranging from 0.743 to 0.989 in identifying all identified major causes of blindness. Further assessment of 4,670 cases in a tertiary eye center also demonstrated that the proposed framework achieved a high identification accuracy rate for different visual impairment diseases compared with that of human graders in a clinical setting. The proposed hierarchical deep learning framework would improve clinical practice in ophthalmology and broaden the scope of service available, especially for people living in areas with a few ophthalmologists.

## Introduction

Eye diseases leading to visual impairment are a significant source of social burden. It is estimated that, as of 2017, 1 billion people were living with vision impairment worldwide, including those with moderate or severe distance vision impairment or blindness caused by unaddressed refractive error (123.7 million), cataract (65.2 million), glaucoma (6.9 million), corneal opacities (4.2 million), diabetic retinopathy (3.0 million), and trachoma (2.0 million), as well as near vision impairment caused by unaddressed presbyopia (826.0 million) (1). In China, the most frequent cause of visual impairment is cataract, which is followed by corneal disease and glaucoma (2, 3). In contrast, age-related macular degeneration and diabetic retinopathy are more prevalent in the United States (4). Early detection and treatment of visual impairment diseases are critical and integral to combating this avoidable blindness worldwide.

A slit-lamp investigation of the ocular surface and retina using manual interpretation is a widely accepted screening tool to detect visual impairment diseases. However, this is highly dependent on the ophthalmologist's clinical experience, which is time-consuming and may have an interobserver variation on the same patient. Automated identification of various visual impairment diseases via slit-lamp photography has benefits such as increased efficiency, reproducibility, and access to eye care. To enable this, artificial intelligence (AI)-based approaches for the identification of visual impairment diseases are greatly needed, especially for people living in areas with a limited number of ophthalmologists.

Recent advances in AI, particularly convolutional neural networks (CNN)-based deep learning algorithms, have made it possible to learn the most predictive disease features directly from medical images given a large dataset of labeled examples (5, 6). Esteva et al. (7) proposed a dermatologist-level classification of skin cancer by fine-tuning a pretrained Inception-v3 network (8). Menegola et al. (9) also conducted experiments comparing training from scratch with fine-tuning of pretrained networks on skin lesion images. Their study showed that fine-tuning of pretrained networks worked better than training from scratch. Setio et al. (10) applied a multi-view CNN to classify points of interest in chest computed tomography as nodules or non-nodules. Similarly, Nie et al. (11) used a three-dimensional CNN on magnetic resonance images to assess the survival of patients suffering from brain tumors.

Because of the fine-grained variability in the appearance of eye lesions, most of the existing eye disease identification methods focused on a single disease type (such as retinopathy and macular diseases) via retinal fundus or optical coherence tomography (OCT) images. Gulshan et al. (12) demonstrated the detection of diabetic retinopathy by fine-tuning a pretrained Inception-v3 network on retinal fundus images. Similarly, Gargeya and Leng (13) performed automated identification of diabetic retinopathy using a ResNet-based architecture. Li et al. (14) adopted an Inception-v3 network to detect glaucomatous optic neuropathy using color fundus images, whereas Burlina et al. (15) applied both a pretrained model and a newly trained from a scratch model for automated grading of age-related macular degeneration from color fundus images. Schlegl et al. (16) and Treder et al. (17) proposed automated detection of macular diseases using OCT images. Long et al. (18) developed a technique for the diagnosis of congenital cataracts. However, their method was focused on images covering the pupil area only; therefore, their algorithm could not detect diseases affecting the peripheral cornea and limbus. To date, there have been few studies diagnosing ocular surface diseases or identifying various disease types simultaneously. Ting et al. (19) proposed a deep learning system for diabetic retinopathy and related eye diseases using retinal images. Fauw et al. (20) proposed an Ensemble-based deep learning framework that could make referral suggestions on retinal diseases by analyzing OCT images. Li et al. (21) presented a workflow for the segmentation of anatomical structures and annotation of pathological features in slit-lamp images, which improved the performance of a deep learning algorithm for diagnosing ophthalmic disorders. As most of these algorithms have been derived from datasets of one or a few ocular diseases, they struggle to detect visual impairment diseases accurately in large-scale, heterogeneous datasets.

To maximize the clinical utility of AI, we developed a hierarchical deep learning framework, which enables early screening and differentiation of a large variety of visual impairment diseases simultaneously in a coarse-to-fine manner. Here, a hierarchical architecture means that multiple classification layers are arranged in a hierarchical way for different levels. To test the feasibility of the proposed framework, we identified eye diseases on two different levels of the eye disease taxonomy. Thereby, in our case, the proposed framework would first perform disease classification for a lower level (i.e., level 1) and then perform a higher-level disease classification (i.e., level 2). Also, algorithm performance was tested against 40 ophthalmologists in a clinic-based dataset. Finally, we performed an observational diagnostic assessment comparison of visual impairment disease screening between the algorithm and the ophthalmologists in a tertiary eye center.

## Materials and Methods

### Datasets

Our dataset came from two major eye centers in China: (i) the Eye and ENT Hospital of Fudan University, Shanghai, and (ii) the Affiliated Hospital of Guizhou Medical University, Guizhou. We used the IM 900 or 600 digital slit-lamp photography system (Haag-Streit, Switzerland) and CR-2 digital non-mydriatic retinal cameras (Canon, Japan). All images were annotated by senior ophthalmologists, where 50% of the proportion included retinal photographs and no images with the dilated pupil were included. Our objective was to provide a fast and cost-effective tool for screening patients with visual impairments. A suspected participant would be referred to a doctor for further assessment, including the dilated examination.

#### Retrospective Dataset

Thirty-two ophthalmologists were invited to grade the images of the retrospective database. During the training process of ophthalmologists, a dataset of 100 images (including 25 corneal disease cases, 25 cataract cases, 25 glaucoma cases, and 25 retinal disease cases) was used for the test. The participants' results were compared with those of two senior corneal specialists (H.G. and J.H.). The participants would not complete the training until they achieved a κ-value of 0.75 or more. A κ-value of 0 indicates that observed agreement is the same as that expected by chance; 1 indicates perfect agreement; 0.75 or more indicates substantial agreement and/or almost perfect agreement. As a result, 20 ophthalmologists were qualified as graders to classify images. Each photograph was reviewed with the same standard and annotated via face-to-face communication between two ophthalmologists. As all 7,100 images from 1,600 patients collected already had original diagnoses recorded in medical charts, graders were asked to review, validate, and classify the images.

#### Prospective Dataset

A total of 4,670 outpatients agreed to receive the test and got their ocular surface slit-lamp photographs taken before their physician visits. Informed consent was obtained from all the participants. A software practitioner participating in this study fed these images as input to the trained deep learning software model. The algorithm generates a probability/confidence score over the classification nodes in a sequential manner, i.e., level by level. If the probability/confidence score of any disease subtype was greater than a predefined threshold, the disease subtype was diagnosed as positive. To quantitatively compare the sensitivity and specificity of our algorithm to that of the other 40 ophthalmologists on the diagnostic task of these cases, receiver operating characteristic (ROC) curves were plotted where each ophthalmologist was asked about the diagnosis on the basis of the images. Thirteen additional cases were also independently collected from clinics for our direct performance test sets.

**Figure 1:**
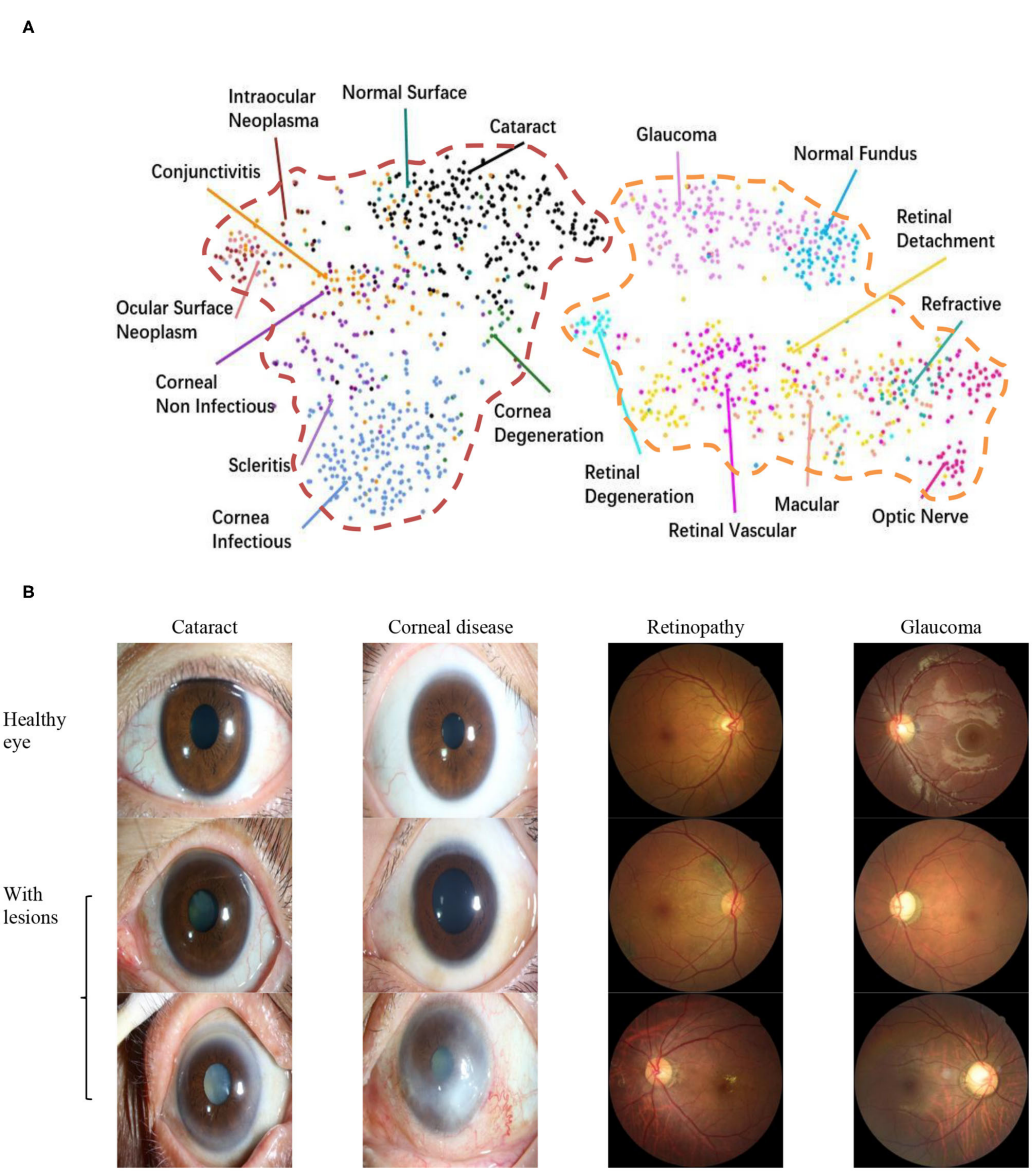
Dataset. **(A)** t-Distributed stochastic neighbor embedding visualization of the collected dataset consisting of 17 major ocular disease classes (100 subtypes), leading to visual impairment, clustered according to deep features generated from the last layer of trained networks. Colored point clouds represent images with different visual impairment diseases. This visualization represents the ability of our method to objectively separate normal patients from early cases of visual impairment diseases for referral. **(B)** Example ocular surface and retinal images for the eye with some common diseases or healthy eye. In this study, the first two levels of the taxonomy consisting of 17 major ocular disease classes (100 subtypes) were used in performance evaluation.

To explore the visual characteristics of different clinical classes, we examined the internal image features learned by the proposed framework using t-distributed stochastic neighbor embedding (22). As demonstrated in Figure 1A, each point represents an eye image projected from the n-dimensional output of the last hidden layer of Inception-v3 backbone into two dimensions. We see clusters of points of the same clinical classes. This visualization represents the ability of our method to objectively separate normal patients from early cases of visual impairment diseases for a referral. Figure 1B shows a few examples of images that demonstrate the visual features using which the proposed hierarchical deep learning framework can identify and make a diagnosis.

### Taxonomy

Inspired by Esteva et al. (7), who defined skin diseases in a tree structure, we adopted a similar approach to define our domain taxonomy structure for eye diseases, taking advantage of fine-grained information embedded within the images. Our taxonomy represented 100 individual diseases hierarchically arranged in a Pie structure. It was derived based on the collected retrospective database with 7,100 images from 1,600 patients by ophthalmologists using a bottom-up procedure: Individual diseases—initialized were defined as leaf nodes, and then were merged on the basis of clinical and visual similarity until the entire structure was connected.

**Figure 2:**
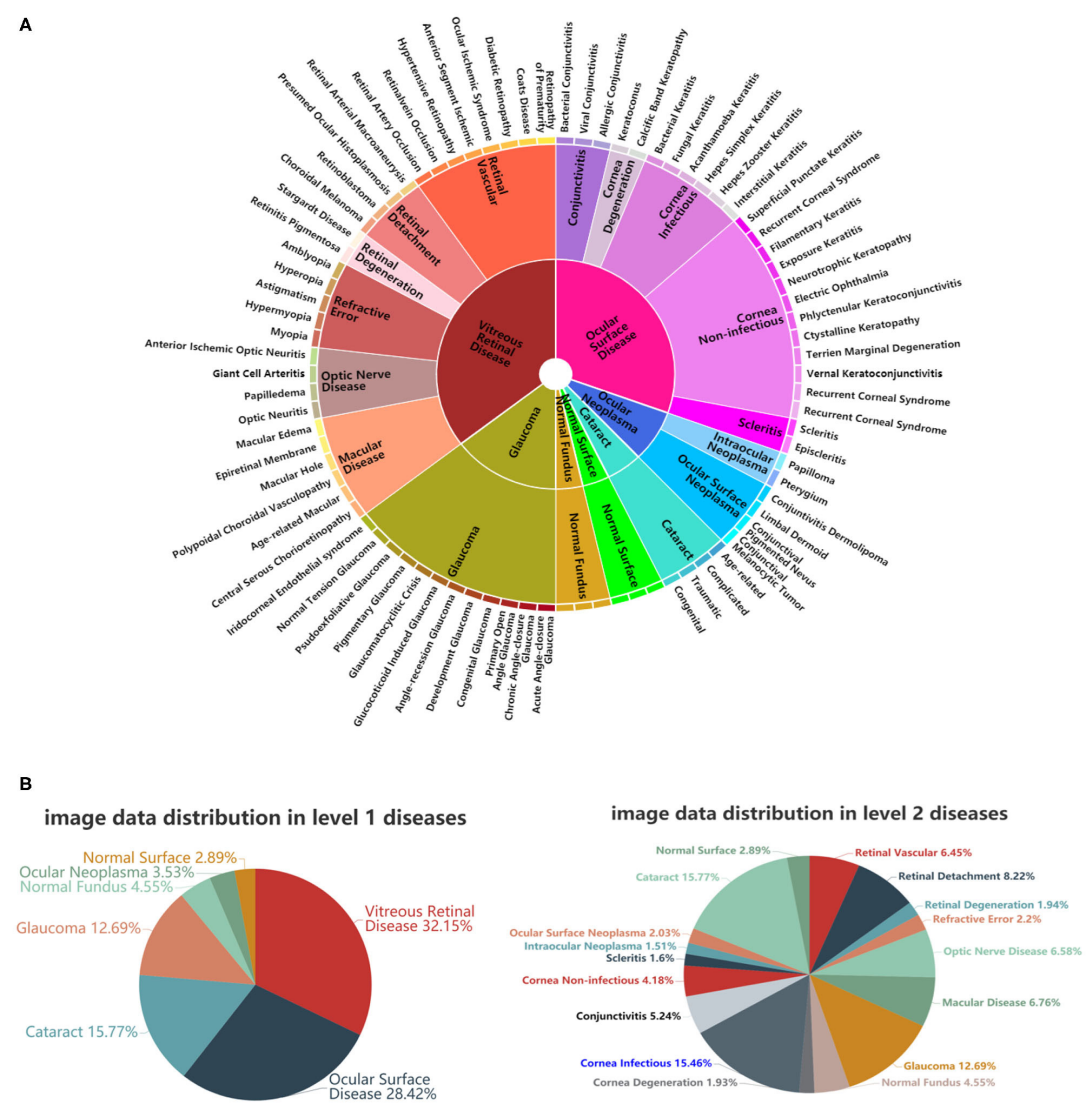
A schematic illustration of the predefined eye disease taxonomy and example test set images. **(A)** Pie-structured eye disease taxonomy. **(B)** Data distribution for the first two levels of diseases.

As shown in Figure 2A, the taxonomy is useful in generating hierarchical training classes that are both well-suited for machine learning classifiers and medically relevant. In this study, the first two levels of the taxonomy were used in performance validation. Figure 2B illustrates the corresponding data distributions. It is worth mentioning that due to insufficient numbers of images for each of the level 3 diseases, we did not perform the level 3 classification. However, the extension to more levels can be implemented via our flexible and extensive framework with sufficient training data.

### Proposed Hierarchical Deep Learning Framework

**Figure 3:**
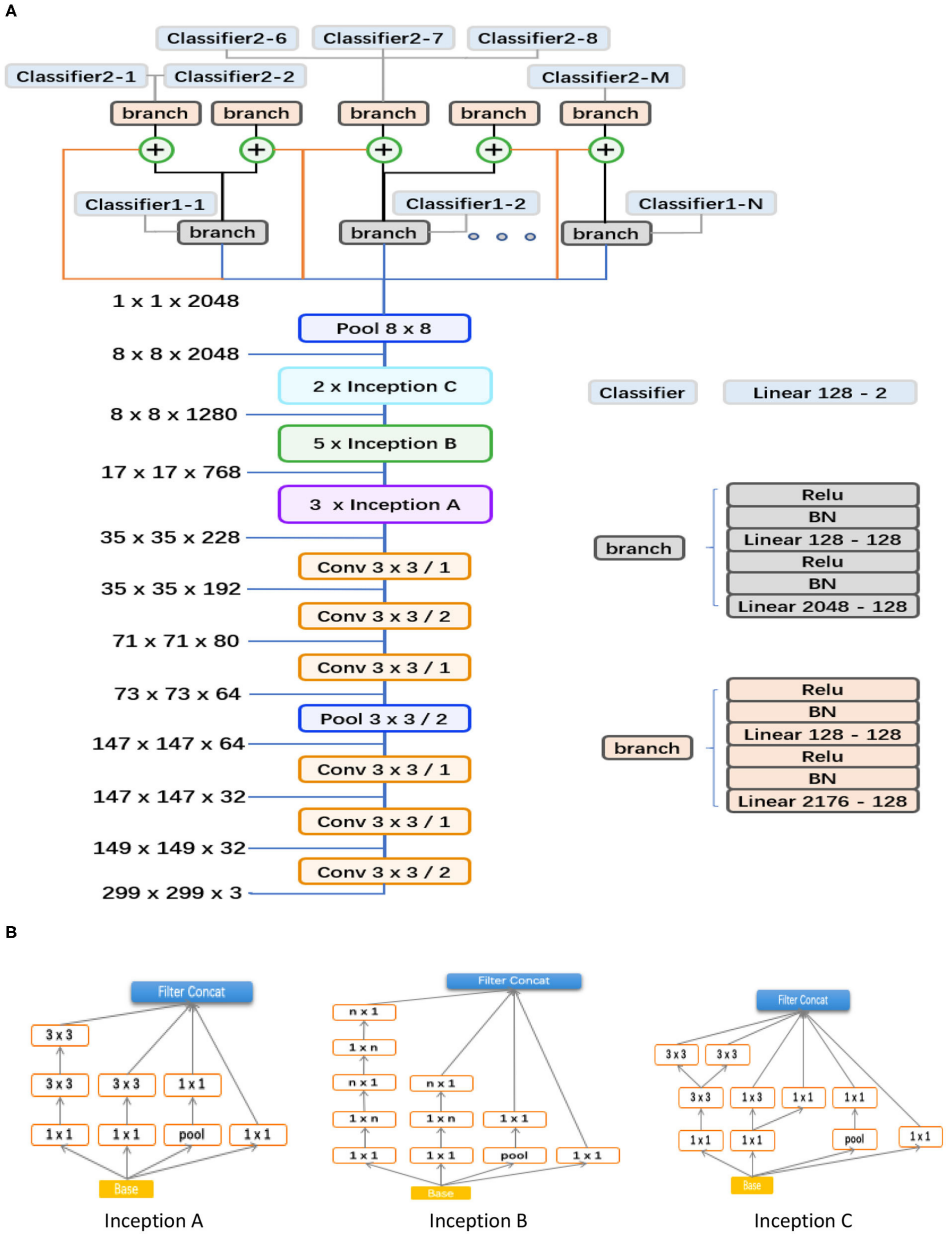
Abstraction of the proposed hierarchical deep learning framework. **(A)** The proposed network architecture based on the feature network of Inception v3 (Conv 3 × 3/2 indicates that a 3 × 3 convolution kernel was used and stride = 2). The corresponding sizes of the input and output for each module are also shown. In our framework, a family of multi-task & multi-label classification layers were used hierarchically to represent various levels of eye diseases. The individual multi-task classifier layer is defined on the basis of a predefined eye disease taxonomy. Here, the data flow in blue indicates that the backbone is directly connected to the branch of level 1; the orange means that the backbone is directly connected to the branch of level 2; the flow in black means connecting from the branch of level 1 to the branch of level 2; and the ⊕ is a feature concatenation operation, where features from the black and orange are superimposed; finally, this 8*8 pooling layer is a global average pooling, which turns the 8*8 feature map into a 1*1 feature map. **(B)** Different spatial factorized Inception modules are presented here. Inception A contains the factorization of the original 5 × 5 convolutions, factorizes general n × n convolutions (*n* = 5 in our study), and has expanded the filter bank outputs.

As shown in Figure 3, the proposed hierarchical deep learning framework is composed of a family of multi-task & multi-label learning classifiers representing different levels of eye disease classification derived from the hierarchical eye disease taxonomy. Here, we used an Inception-v3 CNN as the backbone of the proposed framework, and the final classification layer of the Inception-v3 network was replaced with our novel hierarchical multi-task & multi-label classification layers. Each task branch consists of several stacked fully connected units, hierarchically representing various levels of eye disease classification. As a result, the classification results of lower levels of classifiers can be used as priors for higher levels of classifiers, thereby improving the final classification performance.

We trained the model by minimizing our novel multi-level eye disease–guided loss function consisting of multiple levels of losses. The objective function for two levels can be represented as follows:

(1) $$Loss_{T}=\alpha^{*}loss_{l1}+\left(1-\alpha \right)*loss_{l2}$$

where the term *Loss*_*T*_ is the total loss of the final model, and *loss*_*l*1_ and *loss*_*l*2_ represent the corresponding losses for levels 1 and 2 of eye disease identification, respectively. α is a weight parameter that is used to control the balance between the two losses. For the two levels, α ∈ (0, 0.5), setting more weight for the higher level because the ultimate goal was to classify higher levels of diseases. Through experiments, we found that α = 0.3 performed well (i.e., the loss weight ratio 3:7 between level 1 and 2 classifiers). In this study, we used the sigmoid function for each class instead of the commonly used SoftMax function, for multiple diseases may simultaneously exist. Because of the unbalanced property of data, we applied the focal loss (23) for the loss function of each level, which reduced the impact of data imbalance and made the training focus on hard negatives as well. The focal loss function can be represented as follows:

(2) $$\begin{array}{l} FL\left(p_{t}\right)=-{\left(1-p_{t}\right)}^{\gamma }\;log\left(p_{t}\right) \end{array}$$

where

(3) $$p_{t}=\{\begin{array}{c} p\;if\;y=1\\ 1-p\;otherwise \end{array}\;(3)$$

${\left(1-p_{t}\right)}^{\gamma }$ is a modulating factor of the cross-entropy loss, with a tunable focusing parameter γ ≥ 0, *p* ∈ [0, 1]. During the training process, various data augmentation methods (including horizontal and vertical flipping, color jitter, rotation, etc.) were also applied to all classes independently on-the-fly. It is worth mentioning that the online data augmentation was aimed at increasing the diversity of data for generalization rather than balancing and/or increasing the amount of training data.

Instead of training from scratch, we applied a fine-tuning strategy on a pretrained model using a multi-step retraining strategy. In this study, all images were resized to the size of 299 × 299 since that is the default input size for the Inception-v3 model. We used the Inception-v3 model pretrained on the ImageNet dataset (24) as the initial model and fine-tuned all layers with our dataset. First, the multi-task branches were trained by freezing the backbone's weights for 5 epochs. The Adam optimizer and a learning rate of 0.0001 and epsilon of 0.1 were used. Then, we performed a multi-step retraining strategy. In this strategy, we gradually unfroze the layer weights in steps, with the first few layers being unfrozen last. The learning rates were progressively reduced from 0.0001 to 0.000001, whereas other parameters were kept unchanged. Every step lasted 20 epochs. We used Facebook's PyTorch deep learning framework (25) to train, validate, and test the algorithm networks.

## Results

### Performance Evaluation

Algorithm performance was measured by the area under the ROC curve (AUC) and the accuracy rate. The accuracy rate calculated the percentage of correctly predicted individuals among the whole test set, whereas the ROC curve was generated by plotting the curve of sensitivity against specificity, which can be defined as follows:

(4) $$\begin{array}{l} Accuracy=\frac{TP+TN}{TP+TN+FP+FN} \end{array}$$

(5) $$\begin{array}{l} Sensitivity=\frac{TP}{TP+FN} \end{array}$$

(6) $$\begin{array}{l} Specificity=\frac{TN}{TN+FP} \end{array}$$

where TP, FP, TN, and FN are true positive, false positive, true negative, and false negative rates, respectively. TP and TN represent correctly predicted positives and negatives with respect to the ground truth labels. FP and FN represent incorrectly predicted positives and negatives with respect to the ground truth labels.

**Figure 4:**
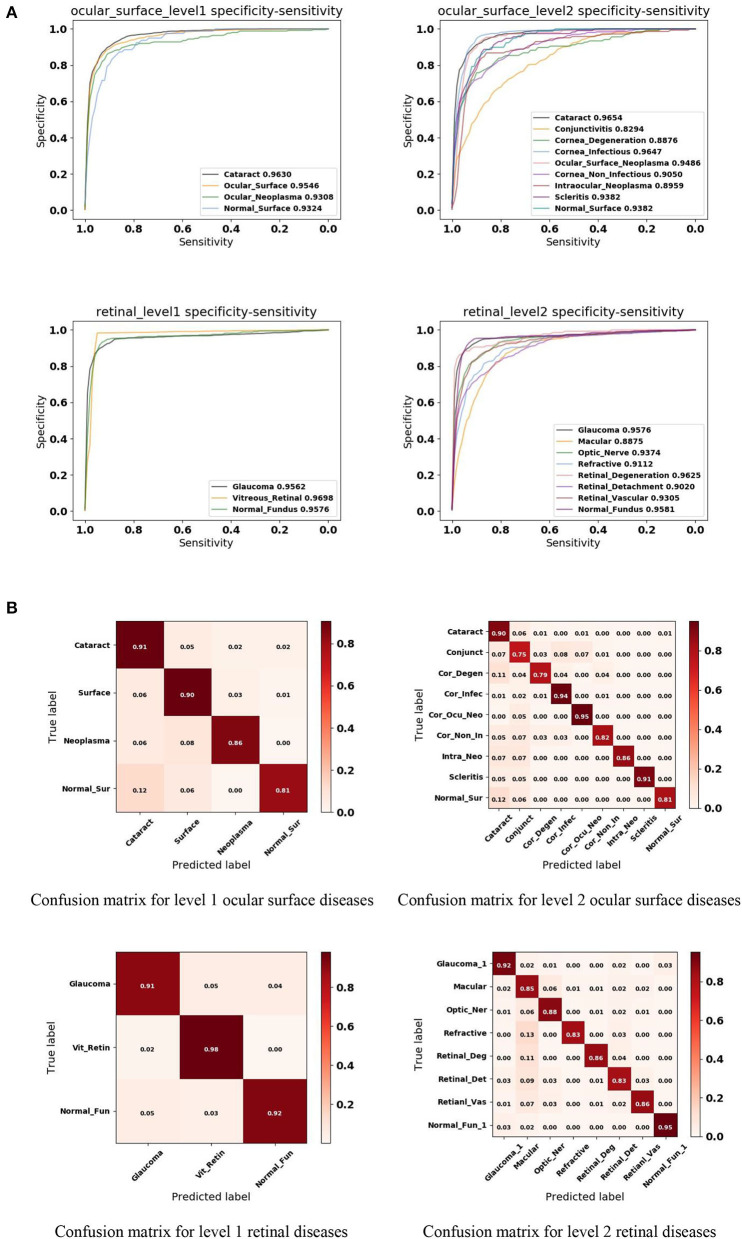
Performance of the proposed hierarchical deep learning framework. **(A)** The mean receiver operating characteristic (ROC) curve for various eye diseases of the first two levels of the eye disease taxonomy. AUC is the area under the ROC curve. **(B)** Confusion matrices for the first two levels of the eye disease taxonomy. Conjunct, Conjunctivitis; Cor_Degen, Corneal_Degeneration; Cor_Infec, Corneal_Infectious; Ocu_Cor_Neo, Ocular_Corneal_Neoplasma; Cor_Non_In, Corneal_Non_Infectious; Intra_Neo, Intraocular_Neoplasma; Normal_Sur, Normal_Surface; Optic_Ner, Optic_Ner; Retinal_Deg, Retinal_Degeneration; Retinal_Det, Retinal_Detachment; Retinal_Vas, Retinal_Vascular; Normal_Fun, Normal_Fundus.

In this study, we applied a 5-fold cross-validation strategy to evaluate the effectiveness of the proposed framework. This strategy randomly divides the entire dataset into five subsets, each containing around 20% of the data. Model training and validation were performed five times. Figure 4A shows that our framework achieved high sensitivity, specificity, and AUC for most of the identified diseases. Figure 4B illustrates the corresponding confusion matrices for disease classification. As shown in level 1 confusion matrices, the CNN model performed extremely well on all three retinal fundus diseases, with an accuracy of 0.91 for glaucoma, 0.98 for vitreoretinal disease, and 0.92 for normal fundus. Meanwhile, the CNN model performed moderately well on all four ocular surface diseases, with an accuracy of 0.91 for cataract, 0.90 for surface disease, 0.90 for neoplasma, and 0.81 for normal surface images. This may be because fundus images contain more discriminative features than do ocular surface images. The model confused normal surface cases with cataract (12.0%) and confused cataract with surface disease (5.0%), neoplasm (2.0%), and normal surface images (2.0%). From these results, we can conclude that it is easy to confuse the normal surface with cataract because of appearance similarities, whereas cataract has more appearance diversity, which can also be confused with other ocular surface diseases and neoplasms. Similar results can be found in level 2 confusion matrices.

**Figure 5:**
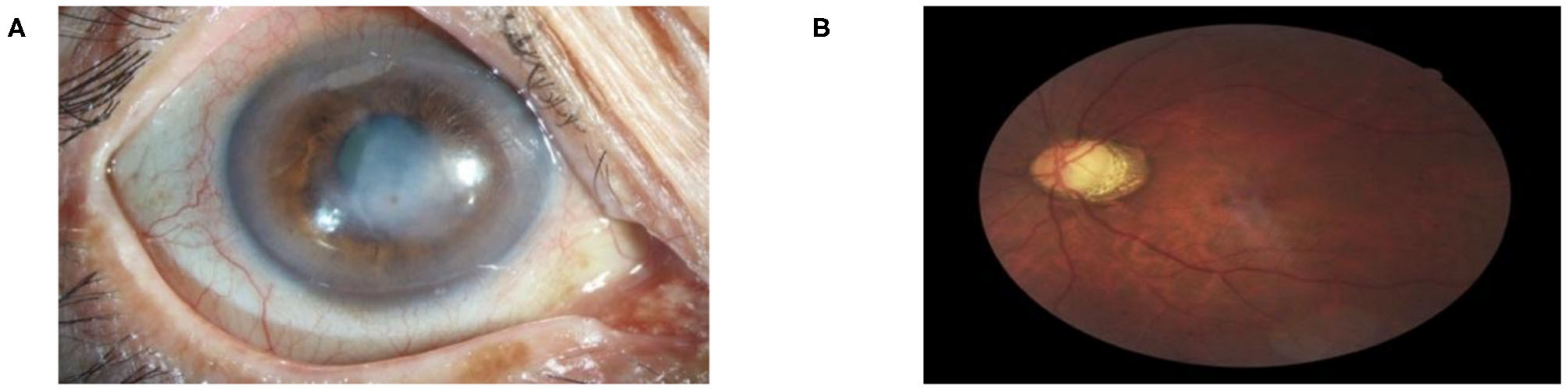
Multi-label diagnostic results. The proposed hierarchical deep learning framework is capable of detecting multiple diseases simultaneously on the same patient: **(A)** cataract with 76.74% and corneal disease with 75.94% confidence and **(B)** glaucoma with 79.04% and retinopathy with 51.01% confidence.

Because of the multi-task & multi-label property of the proposed framework, the trained model is capable of detecting multiple diseases simultaneously on the same patient, reflecting true clinical cases. As illustrated in Figure 5, both cataract and corneal disease were detected simultaneously within a single ocular surface image with 76.74 and 75.94% confidence, respectively. Similarly, both glaucoma and retinopathy were also detected within one retinal image with 79.04 and 51.01% confidence, respectively. It needs to be mentioned here that in this study, if the prediction score was > 50%, the system considered the screening output of the patient with the corresponding disease. In a real-world setting, if the screening output of the patient has one of the diseases listed above, the patient would be referred to a specialist for further diagnosis. Physicians need to consider not only the screening result but also the diagnostic severity of the disease to make clinical decisions for a patient. This was beyond the scope of our study. Our goal was to provide a fast and cost-effective screening tool for patients with visual impairment.

### Comparison Tests

**Figure 6:**
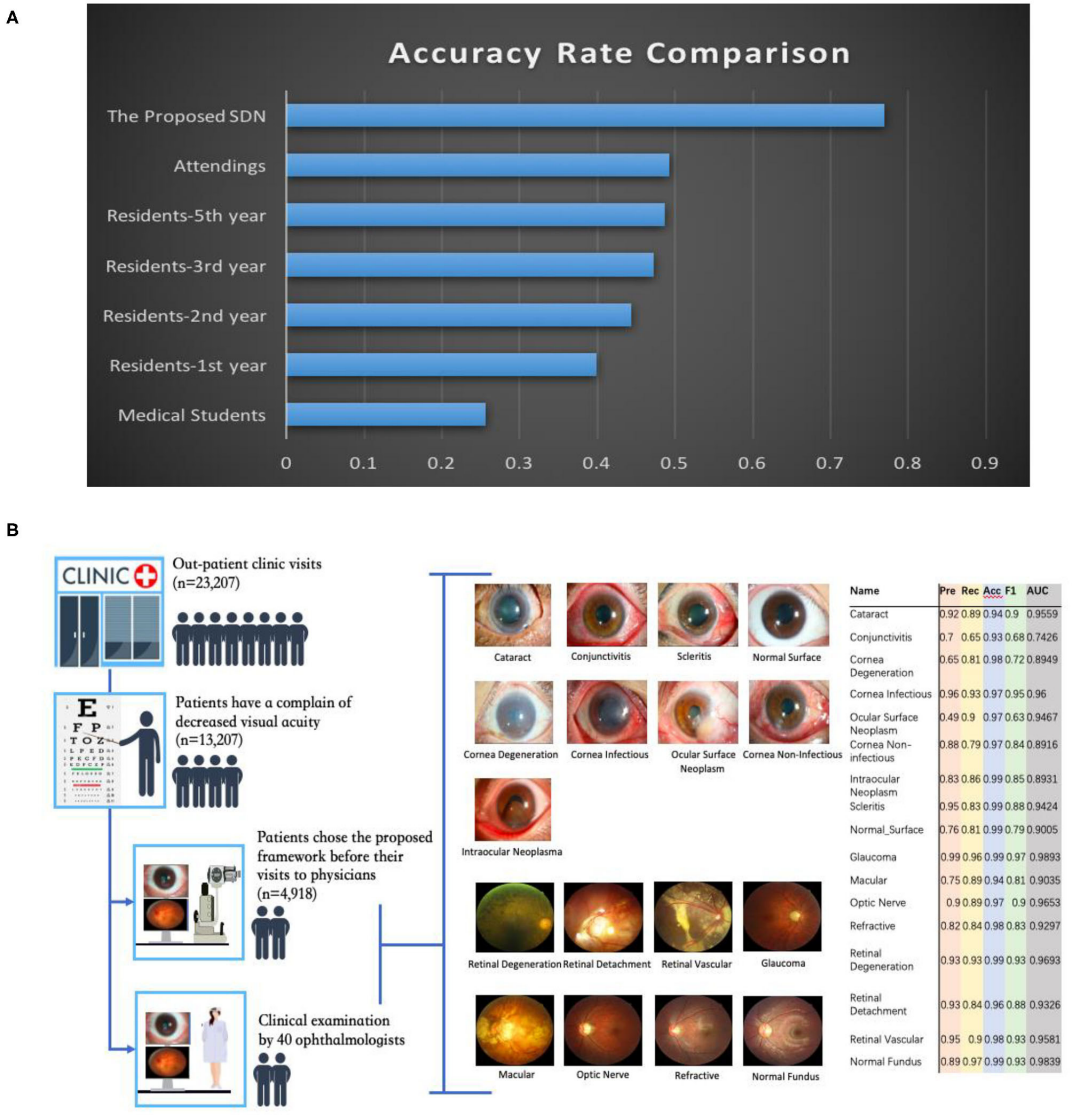
Eye disease classification performance of the proposed hierarchical deep learning framework and ophthalmologists. **(A)** The proposed hierarchical deep learning framework was tested against 40 board-certified ophthalmologists in diagnosing the clinical cases of 13 patients in a real-world setting. For each image, the ophthalmologists were asked to make three diagnoses. The proposed hierarchical deep learning framework outperformed all levels of board-certified ophthalmologists for all cases. **(B)** Clinical application of the proposed hierarchical deep learning framework for visual impairment diseases in a tertiary eye center. Discrepancies between manual grades and the proposed hierarchical deep learning framework results were sent to an independent panel of senior specialists for arbitration.

To both quantitatively and qualitatively demonstrate the effectiveness of the proposed framework, we also compared it with 40 board-certified ophthalmologists in diagnosing clinical cases. The comparison tests used 20 images from 13 patients. The tested diseases include allergic conjunctivitis, dry eye, bacterial conjunctivitis, Mooren's corneal ulcer, keratoconus, fungal keratitis, viral keratitis, scleritis, age-related macular degeneration, cataract, primary angle closure glaucoma, myopia, diabetic retinopathy, and retinal detachment. For this study, each ophthalmologist was asked for the three most likely diagnoses of the patient. This choice of question reflects the actual in-clinic task in which ophthalmologists would decide whether or not to request further examinations. For a fair comparison, the proposed hierarchical deep learning framework also outputs the top three diagnoses with probability/confidence scores. The outcome was considered “correct” when one of the three diagnoses made by the proposed hierarchical deep learning framework or an ophthalmologist included the real diagnosis for the case. Remarkably, the proposed hierarchical deep learning framework outperformed all levels of board-certified ophthalmologists in every case, as shown in Figure 6A (*P* < 0.05 in *t*-test).

In addition, we performed an observational diagnostic assessment comparison between the proposed framework and human graders in a tertiary eye center to determine whether or not the proposed framework can be introduced into visual impairment disease screening. As demonstrated in Figure 6B, 4,670 consecutive patients visiting the Shanghai Eye and ENT Hospital were invited to get their slit-lamp photographs taken before they were checked by their physicians. Discrepancies between manual grades and the proposed hierarchical deep learning framework results were sent to a panel of senior ophthalmologists for arbitration. Our data showed that the proposed hierarchical deep learning framework achieved an acceptable detection accuracy rate for visual impairment disease screening when compared with that of human graders in a clinical setting. The detection AUC of the proposed hierarchical deep learning framework for 17 subclasses in level 2 of visual impairment diseases ranged from 0.743 to 0.989.

**Figure 7:**
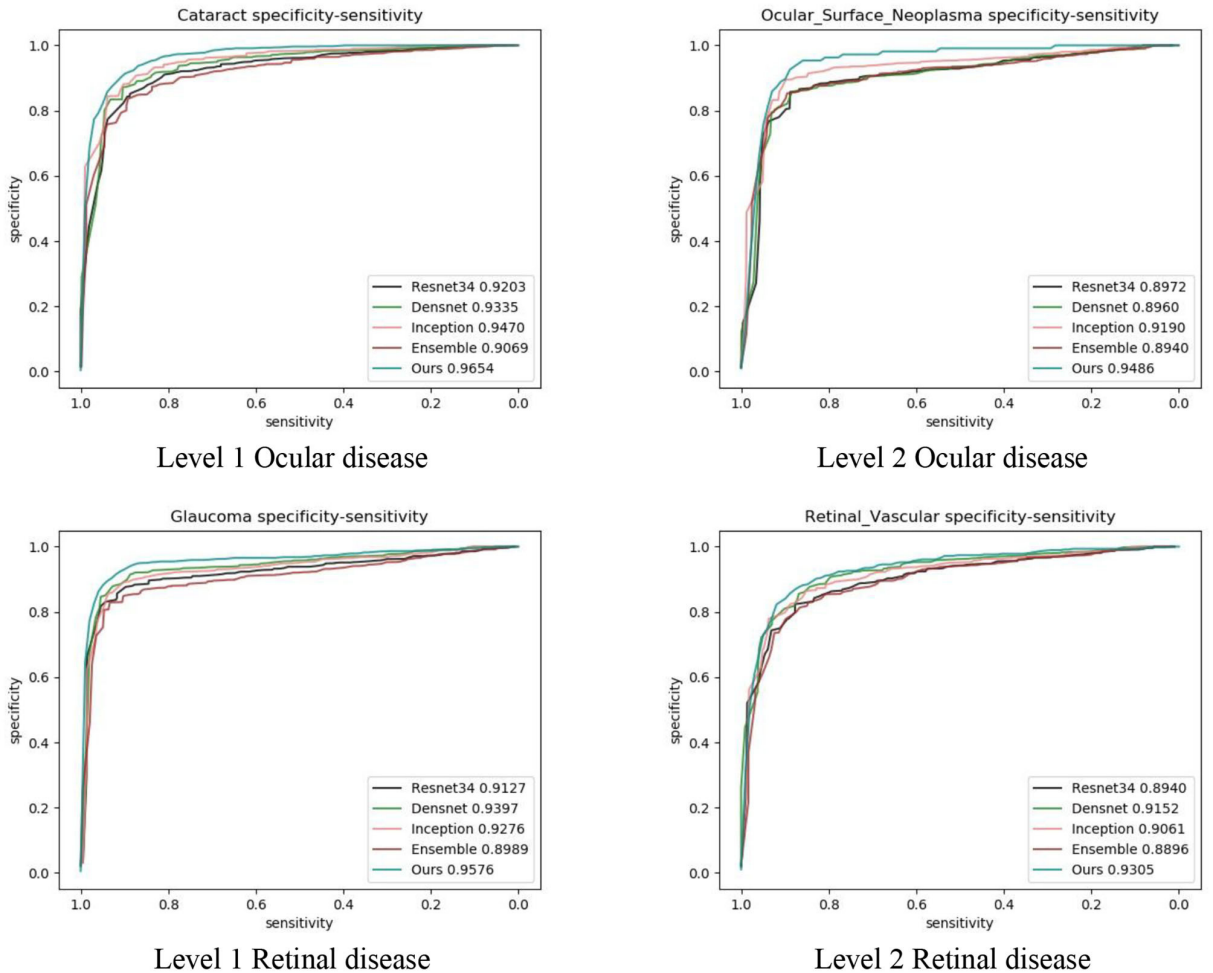
Performance comparison with four deep learning frameworks.

We also compared our algorithm performance with four previously reported methods, namely Inception-v3 (8), ResNet (26), DenseNet (27), and Ensemble (28). The Ensemble model combined all backbone features extracted from the other three models and applied a tree-based classifier for the final classification. To have a fair comparison, all the networks above were also trained as multi-task & multi-label networks but without the proposed hierarchical architecture. To be more specific, the last layers of these networks were replaced with a set of binary classifiers with a flat architecture for each level of the disease classification. As shown in Table 1, the computational costs for both the training and the inference stage were comparable for all models. However, with the proposed hierarchical architecture, our algorithm outperformed all four existing methods in most of the diseases. For example, as shown in Figure 7, for level 1 disease identification—such as glaucoma—our framework achieved AUC 0.958, whereas ResNet, DenseNet, Inception-v3, and Ensemble methods achieved AUC 0.913, 0.940, 0.928, and 0.899, respectively. Similarly, for level 2 disease identification, such as ocular surface neoplasm, our framework achieved AUC 0.949, whereas ResNet, DenseNet, Inception-v3, and Ensemble methods achieved AUC 0.897, 0.896, 0.919, and 0.894, respectively. More detailed comparison results can be found in Tables 2–5.

**Table 1:** Computational cost comparison between the proposed hierarchical deep learning framework and existing deep learning frameworks.

**Computational cost**	**Ours**	**Inception-v3**	**ResNet34**	**DenseNet101**	**Ensemble**
Training (hours)	12.5	11.2	10.0	11.4	11.0
Inference (seconds)	0.097	0.083	0.069	0.075	0.106

**Table 2:** AUC comparison between the proposed hierarchical deep learning framework and existing deep learning frameworks.

	**Ours**	**Inception-v3**	**ResNet34**	**DenseNet101**	**Ensemble**
**Level 1 anterior segment (*****n*** **= no. of images)**					
Cataract (*n* = 1,120)	**0.96**	0.94	0.92	0.93	0.91
Ocular surface (*n* = 2,018)	**0.95**	0.94	0.91	0.93	0.90
Ocular neoplasm (*n =* 251)	**0.93**	0.89	0.89	0.91	0.88
Normal surface (*n =* 205)	**0.93**	**0.93**	**0.93**	0.92	0.90
Weighted average	**0.95**	0.93	0.91	0.93	0.90
**Level 2 anterior segment (*****n*** **= no. of images)**					
Cataract (*n =* 1,120)	**0.97**	0.95	0.92	0.93	0.91
Conjunctivitis (*n =* 372)	**0.83**	0.82	0.81	**0.83**	0.81
Cornea degeneration (*n =* 137)	**0.89**	0.86	0.85	**0.89**	0.83
Cornea infectious (*n =* 1,098)	**0.96**	0.95	0.93	0.94	0.91
Intraocular neoplasma (*n =* 107)	**0.95**	0.92	0.90	0.90	0.89
Cornea non-infectious (*n =* 297)	0.91	0.89	**0.93**	0.88	0.86
Ocular surface neoplasm (*n =* 144)	**0.90**	0.88	0.86	0.87	0.85
Scleritis (*n =* 114)	**0.94**	0.93	0.93	0.93	0.90
Normal surface (*n =* 205)	**0.94**	0.93	**0.94**	0.93	0.91
Weighted average	**0.94**	0.92	0.91	0.91	0.89
**Level 1 retinal disease (*****n*** **= no. of images)**					
Glaucoma (*n =* 901)	**0.96**	0.94	0.91	0.92	0.90
Vitreoretinal disease (*n =* 2,283)	**0.97**	0.95	0.93	0.94	0.92
Normal fundus (*n =* 323)	**0.96**	**0.96**	0.94	0.94	0.92
Weighted average	**0.97**	0.95	0.93	0.94	0.91
**Level 2 retinal disease (*****n*** **= no. of images)**					
Glaucoma (*n =* 901)	**0.96**	0.94	0.91	0.93	0.90
Macular disease (*n =* 480)	**0.89**	0.88	0.85	0.86	0.85
Optic nerve disease (*n =* 467)	**0.94**	**0.94**	0.90	0.91	0.89
Refractive error (*n =* 156)	**0.91**	0.90	0.89	0.89	0.89
Retinal degeneration (*n =* 138)	0.96	**0.97**	0.93	0.96	0.92
Retinal detachment (*n =* 584)	**0.90**	0.89	0.87	0.88	0.85
Retinal vascular disease (*n =* 458)	**0.93**	0.92	0.89	0.91	0.89
Normal fundus (*n =* 323)	0.96	**0.97**	0.93	0.94	0.92
Weighted average	**0.93**	0.92	0.89	0.91	0.88

*Bold value means “Best performance”*.

**Table 3:** Accuracy comparison between the proposed hierarchical deep learning framework and existing deep learning frameworks.

	**Ours**	**Inception-v3**	**ResNet34**	**DenseNet101**	**Ensemble**
**Level 1 anterior segment (*****n*** **= no. of images)**					
Cataract (*n =* 1,120)	**0.93**	0.92	0.9	0.91	0.89
Ocular surface (*n =* 2,018)	**0.92**	0.9	0.88	0.89	0.87
Ocular neoplasm (*n =* 251)	0.96	**0.97**	0.96	0.96	0.95
Normal surface (*n =* 205)	0.98	0.98	**0.99**	0.98	0.98
weighted average	**0.93**	0.92	0.90	0.91	0.89
**Level 2 anterior segment (*****n*** **= no. of images)**					
Cataract (*n =* 1,120)	**0.94**	0.93	0.91	0.92	0.9
Conjunctivitis (*n =* 372)	0.93	0.93	0.93	**0.94**	0.92
Cornea degeneration (*n =* 137)	0.97	0.97	0.97	**0.98**	0.97
Cornea infectious (*n =* 1,098)	**0.97**	0.96	0.94	0.95	0.93
Intraocular neoplasma (*n =* 107)	**0.99**	0.98	0.98	0.98	0.98
Cornea non-infectious (*n =* 297)	0.97	**0.98**	**0.98**	0.97	0.97
Ocular surface neoplasm (*n =* 144)	0.98	**0.99**	0.98	0.98	0.98
Scleritis (*n =* 114)	**0.99**	0.98	0.98	0.98	0.07
Normal surface (*n =* 205)	0.98	0.98	0.98	0.98	**0.99**
Weighted average	**0.96**	0.95	0.94	0.95	**0.90**
**Level 1 retinal disease (*****n*** **= no. of images)**					
Glaucoma (*n =* 901)	**0.96**	0.95	0.93	0.94	0.93
Vitreoretinal disease (*n =* 2,283)	**0.97**	0.96	0.93	0.95	0.92
Normal fundus (*n =* 323)	0.97	**0.98**	0.97	0.97	0.97
Weighted average	**0.97**	0.96	0.93	0.95	0.93
**Level 2 retinal disease (*****n*** **= no. of images)**					
Glaucoma (*n =* 901)	**0.97**	0.96	0.94	0.94	0.93
Macular disease (*n =* 480)	**0.93**	0.92	0.91	0.91	0.9
Optic nerve disease (*n =* 467)	**0.96**	**0.96**	0.95	0.95	0.95
Refractive error (*n =* 156)	0.98	**0.99**	0.98	0.98	0.98
Retinal degeneration (*n =* 138)	**0.99**	**0.99**	0.98	0.98	0.98
Retinal detachment (*n =* 584)	**0.96**	0.95	0.94	0.94	0.93
Retinal vascular disease (*n =* 458)	**0.97**	0.96	0.96	0.96	0.96
Normal fundus (*n =* 323)	**0.99**	0.98	0.98	0.98	0.98
Weighted average	**0.97**	0.96	0.95	0.95	0.94

*Bold value means “Best performance”*.

**Table 4:** Recall comparison between the proposed hierarchical deep learning framework and existing deep learning frameworks.

	**Ours**	**Inception-v3**	**ResNet34**	**DenseNet101**	**Ensemble**
**Level 1 anterior segment (*****n*** **= no. of images)**					
Cataract (*n =* 1,120)	**0.91**	0.9	0.88	0.89	0.86
Ocular surface (*n =* 2,018)	**0.9**	0.89	0.86	0.88	0.85
Ocular neoplasm (*n =* 251)	**0.86**	0.82	0.8	0.84	0.8
Normal surface (*n =* 205)	**0.81**	0.75	0.8	0.75	0.8
Weighted average	**0.90**	0.88	0.86	0.87	0.85
**Level 2 anterior segment (*****n*** **= no. of images)**					
Cataract (*n =* 1,120)	**0.9**	0.89	0.86	0.88	0.85
Conjunctivitis (*n =* 372)	**0.75**	0.73	0.73	0.74	0.73
Cornea degeneration (*n =* 137)	0.78	0.78	0.78	**0.79**	0.77
Cornea infectious (*n =* 1,098)	**0.94**	0.92	0.89	0.9	0.86
Intraocular neoplasma (*n =* 107)	**0.95**	0.9	0.82	0.82	0.86
Cornea non-infectious (*n =* 297)	0.78	0.8	**0.82**	0.8	0.76
Ocular surface neoplasm (*n =* 144)	**0.86**	0.79	0.73	0.76	0.76
Scleritis (*n =* 114)	**0.91**	0.87	0.87	0.87	0.87
Normal surface (*n =* 205)	0.81	0.8	0.81	0.8	**0.82**
Weighted average	**0.88**	0.86	0.84	0.85	0.83
**Level 1 retinal disease (*****n*** **= no. of images)**					
Glaucoma (*n =* 901)	**0.91**	0.89	0.87	0.88	0.86
Vitreoretinal disease (*n =* 2,283)	**0.98**	0.97	0.95	0.96	0.95
Normal fundus (*n =* 323)	0.91	**0.92**	0.88	0.89	0.86
Weighted average	**0.96**	0.94	0.92	0.93	0.92
**Level 2 retinal disease (*****n*** **= no. of images)**					
Glaucoma (*n =* 901)	**0.92**	0.9	0.87	0.88	0.84
Macular disease (*n =* 480)	**0.85**	0.82	0.8	0.79	0.79
Optic nerve disease (*n =* 467)	0.86	**0.88**	0.84	0.84	0.84
Refractive error (*n =* 156)	**0.83**	0.81	0.8	0.77	0.81
Retinal degeneration (*n =* 138)	0.82	**0.86**	0.79	0.82	0.79
Retinal detachment (*n =* 584)	**0.83**	0.79	0.77	0.78	0.75
Retinal vascular disease (*n =* 458)	**0.86**	0.84	0.82	0.84	0.8
Normal fundus (*n =* 323)	0.89	**0.91**	0.88	0.89	0.86
Weighted average	**0.87**	0.86	0.83	0.83	0.81

*Bold value means “Best performance”*.

**Table 5:** Precision comparison between the proposed hierarchical deep learning framework and existing deep learning frameworks.

	**Ours**	**Inception-v3**	**ResNet34**	**DenseNet101**	**Ensemble**
**Level 1 anterior segment (*****n*** **= no. of images)**					
Cataract (*n =* 1,120)	**0.88**	0.85	0.81	0.84	0.8
Ocular surface (*n =* 2,018)	**0.96**	0.94	0.93	0.93	0.92
Ocular neoplasm (*n =* 251)	**0.7**	0.68	0.67	**0.7**	0.63
Normal surface (*n =* 205)	0.59	**0.75**	0.71	**0.75**	0.71
Weighted average	**0.90**	0.88	0.86	0.88	0.85
**Level 2 anterior segment (*****n*** **= no. of images)**					
Cataract (*n =* 1,120)	**0.91**	0.89	0.86	0.87	0.85
Conjunctivitis (*n =* 372)	**0.67**	0.66	0.63	0.65	0.61
Cornea degeneration (*n =* 137)	0.64	0.64	0.6	**0.7**	0.61
Cornea infectious (*n =* 1,098)	**0.95**	0.94	0.92	0.93	0.92
Intraocular neoplasma (*n =* 107)	**0.68**	0.62	0.62	0.62	0.58
Cornea non-infectious (*n =* 297)	0.88	0.9	**0.91**	0.89	0.87
Ocular surface neoplasm (*n =* 144)	**0.99**	0.98	0.98	**0.99**	0.97
Scleritis (*n =* 114)	**0.98**	0.97	0.96	**0.98**	0.95
Normal surface (*n =* 205)	0.87	0.92	**0.93**	0.92	**0.93**
Weighted average	**0.88**	0.87	0.85	0.86	0.84
**Level 1 retinal disease (*****n*** **= no. of images)**					
Glaucoma (*n =* 901)	**0.94**	0.91	0.86	0.88	0.86
Vitreoretinal disease (*n =* 2,283)	**0.98**	0.96	0.95	0.96	0.94
Normal fundus (*n =* 323)	0.91	0.87	0.93	0.91	**0.95**
Weighted average	**0.96**	0.94	0.93	0.93	0.92
**Level 2 retinal disease (*****n*** **= no. of images)**					
Glaucoma (*n =* 901)	**0.95**	0.93	0.89	0.9	0.88
Macular disease (*n =* 480)	**0.7**	0.66	0.62	0.64	0.62
Optic nerve disease (*n =* 467)	0.82	**0.85**	0.8	0.81	0.79
Refractive error (*n =* 156)	0.96	0.96	**0.99**	0.96	0.96
Retinal degeneration (*n =* 138)	0.82	**0.86**	0.79	0.79	0.76
Retinal detachment (*n =* 584)	**0.9**	0.87	0.84	0.87	0.81
Retinal vascular disease (*n =* 458)	**0.93**	0.89	0.89	0.89	0.86
Normal fundus (*n =* 323)	0.91	0.92	**0.93**	0.92	0.92
Weighted average	**0.88**	0.86	0.84	0.85	0.82

*Bold value means “Best performance”*.

### Saliency Maps

**Figure 8:**
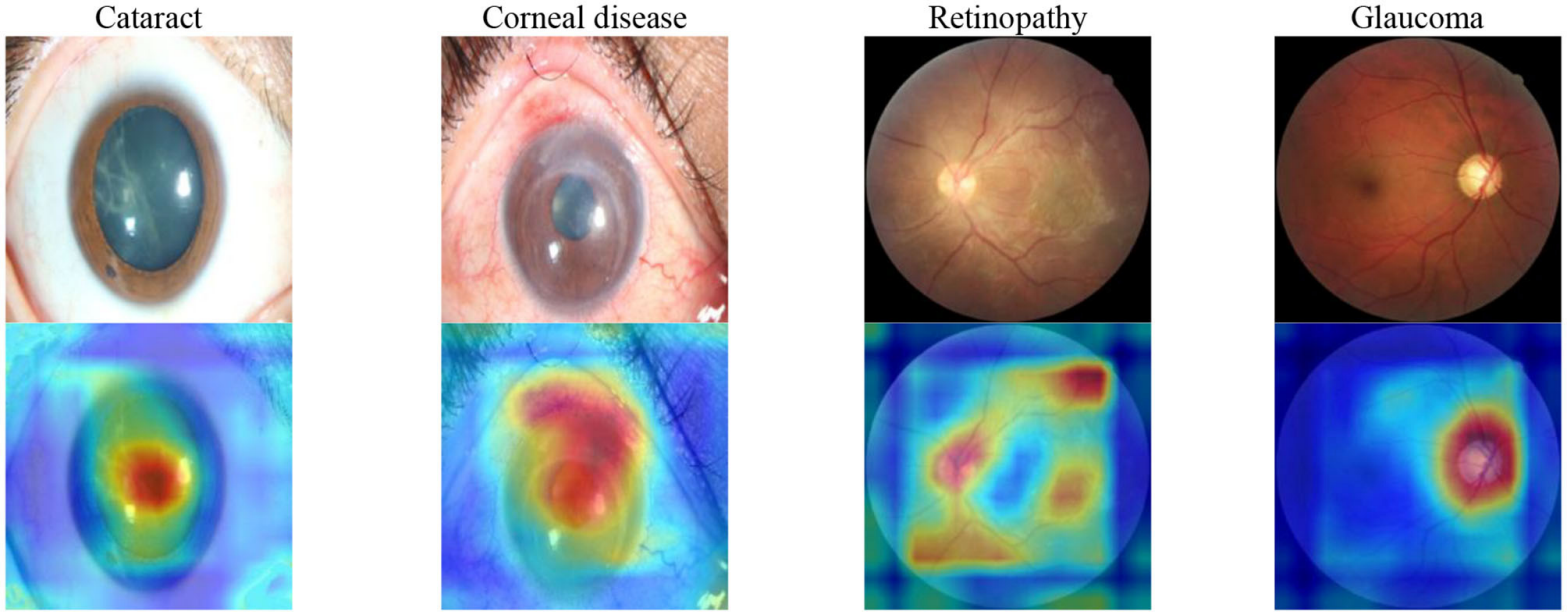
Saliency maps for images with various common visual impairment diseases. These visualizations are generated automatically, locating regions for closer examination after a patient is seen by a consultant ophthalmologist. The bluer the color, the lower the attention of the model; the redder the color, the higher the attention of the model. Visualization maps are generated from deep learning features.

To show the interpretation of the proposed framework, we also created heatmaps via the gradient-weighted class activation mapping (Grad-CAM) algorithm (29), which can produce visual explanations for CNN-based deep learning models. Grad-CAM uses the gradient information flowing into the last convolutional layer to understand the importance of each neuron for a decision of interest, thereby highlighting the important regions in the image for prediction. It first computes the gradient of the score for a given class with respect to feature maps of a convolutional layer. Then, these gradients are average-pooled to obtain the neuron importance weights. Finally, the coarse heatmap for a given class is generated via a weighted combination of forward activation maps followed by a ReLU function. As illustrated in Figure 8, the generated heatmaps helped indicate the potential corneal lesion regions for further examination, thereby establishing prediction trust and interpretation for physicians.

## Discussion

In this study, we demonstrated the effectiveness of the proposed hierarchical deep learning framework in identifying most causes of visual impairment diseases worldwide. Training the proposed hierarchical deep learning framework on eye images captured using commonly available equipment, we outperformed the performance of 40 board-certified ophthalmologists on 13 clinical cases. Further assessment of 4,670 cases in a tertiary eye center also demonstrated that the proposed framework achieved a high identification accuracy rate for different visual impairment diseases compared with that of human graders in a clinical setting.

Although we acknowledge that the clinical impression and diagnosis by an ophthalmologist are based on contextual factors beyond the visual inspection of the eye, the ability to classify eye images with the accuracy of a board-certified ophthalmologist has the potential to profoundly expand access to vital medical care. It has the potential to aid the delivery of eye disease screening in developed and developing countries in a manner that is inexpensive, efficient, and easily accessible. It can also be used to provide eye care guiding services in communities and assist doctors in diagnosing visual impairment diseases.

To validate this technique across the full distribution and spectrum of visual impairment diseases encountered in a clinical setting, further research is necessary to evaluate performance in a large community screening setting. This method is primarily constrained by data and can be validated for more visual conditions if sufficient training examples are provided.

In this study, we applied multiple train–test splits via a 5-fold cross-validation where we randomly divided the entire image dataset into five subsets. Splitting data with respect to patients instead of images is indeed a better strategy; however, the dataset we had did not contain user identification information after data anonymization. We added this as a limitation of our study and would maybe explore it as future work. We would also conduct further experiments with publicly available datasets (such as EyePACS; Kaggle) as one of the future works. In the future, it may also be important to investigate different types of common patient metadata, such as genetic factors, patient history, and other clinical data that may influence a patient's risk of visual impairment diseases. Adding this information to the classification model may yield insightful information outside of strictly imaging information, potentially enhancing the diagnostic accuracy.

## Data Availability Statement

The original contributions presented in the study are included in the article/supplementary material, further inquiries can be directed to the corresponding author/s.

## Ethics Statement

The studies involving human participants were reviewed and approved by the Institutional Review Board of the Shanghai Eye and EENT Hospital (EENTIRB20170607). The patients/participants provided their written informed consent to participate in this study.

## Author Contributions

XL and JH: conception and design. XL, JH, and JC: administrative support. JH, LG, JX, YL, and XS: provision of study materials or patients. JH, XL, HG, ZY, and JL: collection and assembly of data. YG: data analysis and interpretation. All authors: manuscript writing and final approval of manuscript.

## Conflict of Interest

BP and JC are employed by the company Complete Genomics Inc. The remaining authors declare that the research was conducted in the absence of any commercial or financial relationships that could be construed as a potential conflict of interest.
